# General Strategy toward Laser Single-Step Generation of Multiscale Anti-Reflection Structures by Marangoni Effect

**DOI:** 10.3390/mi13091491

**Published:** 2022-09-08

**Authors:** Jingbo Yin, Huangping Yan, Gesang Dunzhu, Rui Wang, Shengzhu Cao, Rui Zhou, Yuanzhe Li

**Affiliations:** 1 School of Aerospace Engineering, Xiamen University, Xiamen 361005, China; 2 Shenzhen Research Institute of Xiamen University, Shenzhen 518000, China; 3 Science and Technology on Vacuum Technology and Physics Laboratory, Lanzhou Institute of Physics, Lanzhou 730000, China

**Keywords:** anti-reflection, multiscale structure, Marangoni effect, anti-glare, femtosecond laser

## Abstract

The anti-reflection of transparent material surfaces has attracted great attention due to its potential applications. In this paper, a single-step controllable method based on an infrared femtosecond laser is proposed for self-generation multiscale anti-reflection structures on glass. The multiscale composite structure with ridge structures and laser-induced nano-textures is generated by the Marangoni effect. By optimizing the laser parameters, multiscale structure with broadband anti-reflection enhancement is achieved. Meanwhile, the sample exhibits good anti-glare performance under strong light. The results show that the average reflectance of the laser-textured glass in the 300–800 nm band is reduced by 45.5% compared with the unprocessed glass. This work provides a simple and general strategy for fabricating anti-reflection structures and expands the potential applications of laser-textured glass in various optical components, display devices, and anti-glare glasses.

## 1. Introduction

Transparent material is widely used in various optical windows [1,2], device screens [3], photoelectric equipment [4,5], and optical elements [6] because of its special optical properties. However, due to the refractive index mismatch of the two media, there is still reflection on the surface of the transparent material. The reflected light will result in interference signals in the precision optical system. In addition, the reflected light from various display devices will produce glare and degrade the user experience. More seriously, the dashboard, navigation instrument, rearview mirror, and front windshield, as the main sources of information for drivers during the driving process, are prone to producing glare on the interface, which may cause a great safety hazard [7]. Therefore, it is of practical significance to explore efficient and simple preparation methods for anti-reflection and anti-glare surfaces.

Preparing microstructure with light-trapping function on the material surface is considered an effective anti-reflection method [7,8]. This strategy has great anti-reflection effects on non-transparent materials such as silicon [9] and metal [8,10]. After a long period of evolution, organisms in nature have a variety of functional surfaces to adapt to challenging environmental changes [11,12,13,14]. Studies have shown that the subwavelength structure with moth-eye effect has advanced anti-reflection performance [15,16,17]. At present, subwavelength structures are often fabricated by the sol-gel method [18], nanoimprinting [15,17,19], electron beam lithography [20,21], laser printing [22,23,24], etc. Moreover, researchers have found that the subwavelength LIPSS (laser-induced periodic surface structures) could be induced on the material surface by adjusting the ultrafast laser polarization shape and the external atmosphere [25,26,27]. Papadopoulos et al. [16] used circularly polarized ultrashort laser pulses to induce the nano-columnar LIPSS, which had a moth-eye effect on fused silica. The average reflectance at 400–1200 nm is reduced from approximately 7.5% to 5%. Additionally, the reflectance is further reduced after double-sided laser treatment. The moth-eye effect depends on the refractive index gradient of the subwavelength structure, which requires the shape and density of the subwavelength structure to meet specific conditions. Therefore, the large-scale preparation of subwavelength structures with a moth-eye effect by the above methods is complicated.

In recent years, various types of micro-nanostructures inspired by nature have also attracted extensive attention [7,28,29]. The moth-eye effect and light trapping effect of multiscale structures can not only reduce the Fresnel reflection loss on the material surface but also change the direction of the incident light [30]. Niu et al. obtained butterfly wing replicas with excellent anti-reflection properties by the sol-gel method and selective etching, and the anti-reflection ability was due to the multiple scales of the structure size and multiple anti-reflection mechanisms [31]. Yu et al. prepared micro-nanostructures by wet etching and plasma etching and demonstrated that the dual-scale structure has excellent anti-reflection performance [32]. Hsueh et al. formed a hybrid structure including pyramids, etched holes, and inverted pyramidal cavities on silicon by three-step chemical etching to obtain a low reflectance surface [33]. Meanwhile, compared with the single-scale moth-eye structure, the multiscale structure has lower requirements for the nanoscale structure characteristics, which reduces the difficulty of preparing nanostructures. However, multiscale structures are usually fabricated step by step with different levels, and the preparation process is relatively complicated.

During laser irradiation, high laser energy density drives phase explosion in the crater center [34], which can expel the neighboring melt sideways. Feng et al. presented that laser pulses with a high repetition frequency caused a cumulative thermal effect, which was beneficial to keeping the material in a molten state [35]. Meanwhile, the hot and cold zones on the material surface cause a difference in tension gradient. It will lead to the phenomenon of spontaneous mass transfer caused by the Marangoni effect [36]. The nano-textures induced by laser irradiation show obvious laser polarization and wavelength dependence [37,38]. Nevertheless, the multiscale structure has low requirements for nano-textures, which can reduce the dependence on specific laser polarization and wavelength. Therefore, by proper utilization of the ridge produced by Marangoni effect combined with the nano-textures induced by laser processing, a multiscale structure with multiple anti-reflection effects can be fabricated.

In this work, the Marangoni effect was adopted in femtosecond laser processing to directly fabricate a micro-nano hybrid structure combining ridge structures and nanotextures in a single step. Multiscale structures showed high controllability and broadband anti-reflection enhancement. Meanwhile, the glare is obviously reduced when the laser-textured glass is under strong light. The proposed single-step preparation method is simple in process, high in applicability, free of any chemical components, environmentally friendly, and has excellent application prospects.

## 2. Experimental Setup

### 2.1. Materials and Instruments

The material selected in this experiment was inorganic glass (B270, SCHOTT, Jena, Germany) with a size of 30 mm × 30 mm × 3.2 mm.

A multiscale structure was fabricated by the linearly polarized infrared laser (wavelength: 1028 nm, pulse duration: 290 fs, maximum power: 15 W, maximum pulse repetition rate: 75 KHz) and the linearly polarized ultraviolet laser (wavelength: 343 nm, pulse duration: 290 fs, maximum power: 15 W, maximum pulse repetition rate: 75 KHz), respectively.

### 2.2. Machining Strategy of the Marangoni Effect

**Figure 1:**
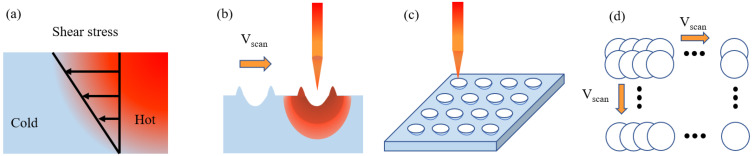
Schematic diagram of the Marangoni effect and laser processing dot matrix structure. (**a**) Marangoni convection; (**b**) Marangoni effect in laser processing; (**c**) laser processing method; (**d**) laser scanning dot matrix.

During the laser processing, part of the material in the laser irradiated area will remain in a molten state, and there will be a significant temperature difference between the irradiated area and the nearby non-irradiated area. Usually, the surface tension of the liquid decreases with the increase in temperature, so shear stress will be generated in the area of the cold and hot interface of the material [36], as depicted in Figure 1a. The repetition frequency of the pulse laser is 25 KHz, which is beneficial to the accumulation of thermal effect in the laser irradiation area. Consequently, the material surface causes Marangoni convection, as shown in Figure 1b. In selective laser sintering processes, non-planar surfaces caused by the Marangoni effect are often considered as machining defects, known as the balling effect [39,40]. However, with the optimization of the laser processing parameters supported by theoretical analysis, the morphology can be easily regulated into the desired structure. Therefore, by proper utilization of the Marangoni effect, a ridge-shaped structure with a size smaller than the light spot can be obtained at the edge of the light spot. Meanwhile, many nanoparticles will be induced in the laser ablation area. Multiscale structures with light trapping effects and moth-eye effects can be achieved by coupling ridge-shaped structures with nanoparticles. To obtain more abundant ridge structures, a laser scanning dot matrix was designed (Figure 1c,d).

### 2.3. Characterization

A field emission scanning electron microscope (SEM, SUPRA55 SAPPHIRE, Carl Zeiss AG, Oberkochen, Germany) and a laser confocal microscope (LCM, VK-X1000, KEYENCE, Osaka, Japan) were used to characterize the surface morphology of multiscale structure samples. The reflectance spectra of the samples at 300–800 nm were measured by a spectrophotometer (Lambda 1050+, PerkinElmer, Waltham, MA, USA) with an integrating sphere accessory.

## 3. Results and Discussion

### 3.1. Anti-Reflection Mechanism

When light propagates between two media, reflected light due to refractive index mismatch is inherent and cannot be completely eliminated. The micro-nano structure can effectively reduce the surface reflection, and its mechanism is mainly determined by the relationship between the structure size and the wavelength of the incident light [7]. 

**Figure 2:**
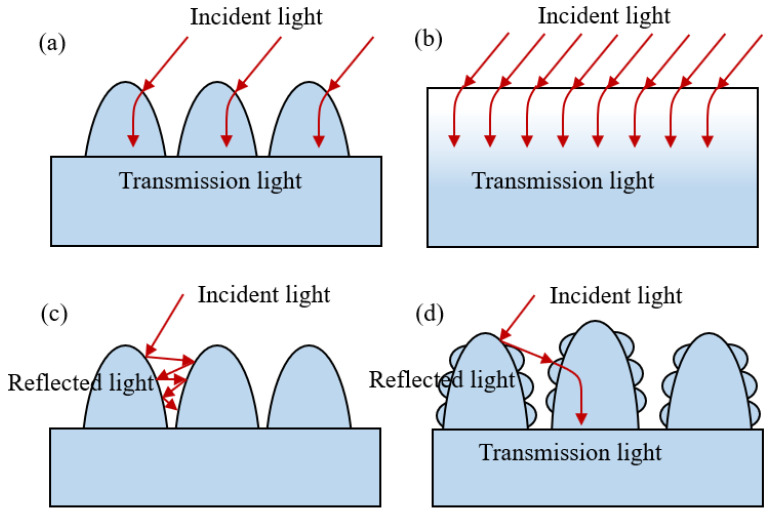
Schematic diagram of light acting on different structure sizes. (**a**,**b**) subwavelength structure; (**c**) micrometer structure; (**d**) multiscale structure.

When the structure is a subwavelength structure, whose size is smaller than the wavelength of the incident light, the interaction between light and structure is shown in Figure 2a. At this time, the incident light cannot completely distinguish the structure form, and the structure can be equivalent to a graded refractive index layer. In this way, the reflected light caused by the sharp change in the refractive index between the two media is reduced, resulting in a moth-eye effect, as shown in Figure 2b. When the spacing and depth between structural surface topographies have the same size constraint as the wavelength of incident light, the light will be reflected multiple times inside the structure. This will produce a light trapping effect, as shown in Figure 2c. In Figure 2d, when the structure is a micro-nano hybrid structure with a small size span, it is called a multiscale structure. The multiscale structure has both a light trapping effect and a moth-eye effect, which is more conducive to improving the anti-reflection ability of the surface. The shape characteristics are less critical than those of the single-scale subwavelength structure because it does not rely solely on the moth-eye effect. 

**Figure 3:**
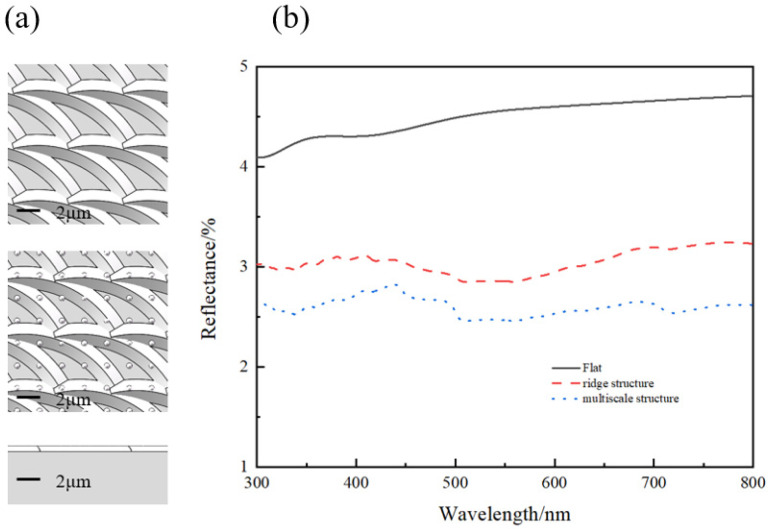
Simulation diagram of the anti-reflection effect of a multiscale structure. (**a**) simulation model, (**b**) reflectance spectrum.

Then, in order to investigate the anti-reflection performance of multiscale structures, the simulation analysis was first carried out. Figure 3a presents the established model, in which the strip-like structure represents ridge structures produced by the Marangoni effect, and the spherical-like structures represent the nanoparticles induced by laser. The reflection spectrum of the flat, ridge structure, and multiscale structure is displayed in Figure 3b, respectively. The anti-reflection performance of multiscale structures is the best. This result indicates that the ridge structure can form a forward-coupled multiscale structure with nanoparticles, which has better anti-reflection properties than a single structure.

### 3.2. Preparation of Multiscale Structure

**Figure 4:**
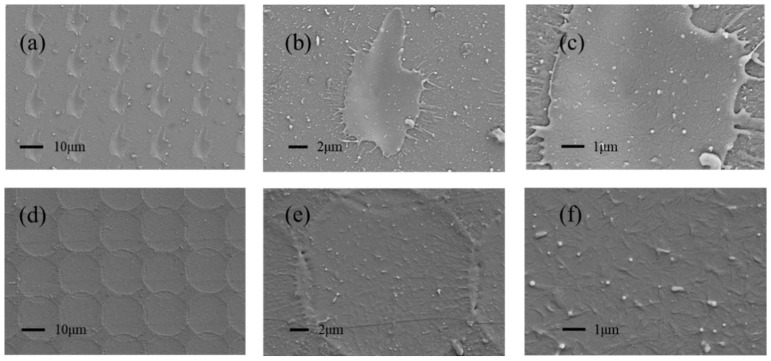
SEM images of the glasses textured by different lasers. (**a**–**c**) ultraviolet femtosecond laser, (**d**–**f**) infrared femtosecond laser.

In Figure 4, SEM was used to characterize the surface processed by the ultraviolet and infrared femtosecond laser. The results show that the spot on the sample surface irradiated by the ultraviolet laser is not a regular circle, as shown in Figure 4a. The Marangoni effect at the edge of the light spot is weak, and there are no obvious ridge structures, as shown in Figure 4b. The spot formed by the infrared femtosecond laser on the sample surface is a regular circle, as depicted in Figure 4d. Additionally, there is an obvious ridge structure at the edge of the light spot, as shown in Figure 4e. Meanwhile, comparing Figure 4c,f, the number of nanoparticles induced by the two lasers is similar, but the line-like nanotextures induced by the ultraviolet laser are fewer. It can be inferred that the infrared laser has greater advantages in the generation of multiscale structures.

**Figure 5:**
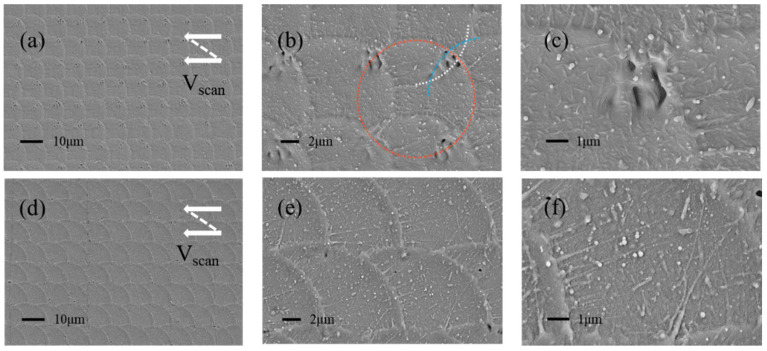
SEM images of infrared laser-textured glass at different laser energy densities. (**a**–**c**) the laser energy density is 57.3 J/cm^2^, (**d**–**f**) the laser energy density is 95.5 J/cm^2^.

The laser energy density affects the morphology of ridge structures as well as the abundance of nanostructures. Figure 4 shows that the spot of the infrared laser is about 20 μm. When the scanning distance is 10 μm, the overlap between the spots increases. The effect of laser energy density on the morphology of ridge structures generated by single light spots and ridge structures generated by overlapping can be investigated at the same time. Figure 5 shows the SEM images of infrared laser-textured glass at different laser energy densities. When the laser energy density decreases, the diameter of the laser spot decreases, and there are plenty of micropores and cracks inside the spot, as shown in Figure 5a,d.

In Figure 5b, as the laser scans, the light spots overlap, and the white, blue, and red arcs represent the ridge structures generated successively. The newly formed light spot will ablate the existing ridge and form a new ridge on its edge by the Marangoni effect. When the laser energy density is 57.3 J/cm^2^, the existing ridge structures are unevenly ablated, and cracks and micropores are formed at the ridge. At the same time, the material flow caused by the Marangoni effect is weakened. Due to the higher height of the incompletely ablated ridge, the light spot formed later cannot completely cover the existing ridge, and finally, there are many cracks and micropores inside the light spot. When the laser energy density is 95.5 J/cm^2^, the ablation of the existing ridge is more uniform, so the ablated ridge is flatter. Moreover, the Marangoni effect is significantly enhanced, and the molten material can completely cover the existing ridges. Therefore, there are almost no micropores and cracks where the ridges overlap, as depicted in Figure 5e. In addition, the increase in the laser energy density also leads to more nanoparticles and line-like nanotextures, as shown in Figure 5c,f. 

**Figure 6:**
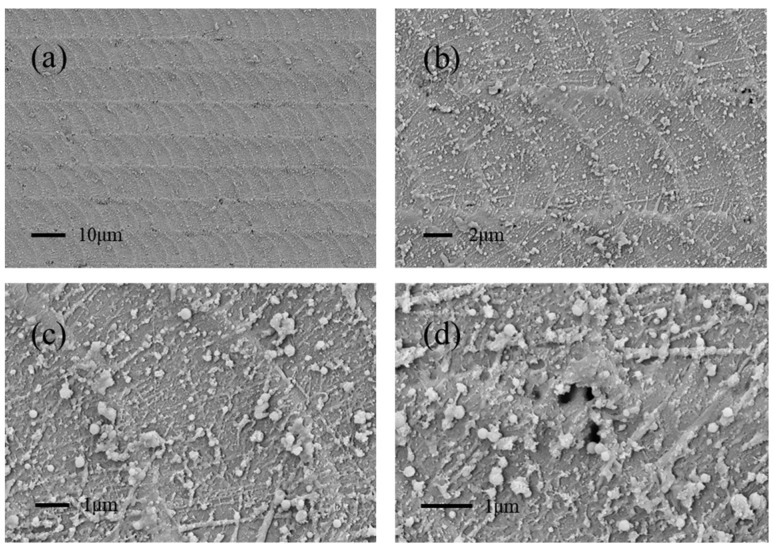
SEM image of the laser-textured glass with different magnifications when the infrared laser scanning distance is 5 μm. (**a**) 1000 times magnification, (**b**) 4000 times magnification, (**c**) 10,000 times magnification, and (**d**) 15,000 times magnification.

To increase the density of ridges, the scanning distance is further reduced to 5 μm, as shown in Figure 6. The light spots can be perfectly overlapped, and almost no micro-holes or cracks are generated. Meanwhile, the laser-induced nanoparticles and line-like nanotextures were more abundant with the increase of spot overlap. Overall, the resulting multiscale structure exhibits morphological diversity at each scale.

**Figure 7:**
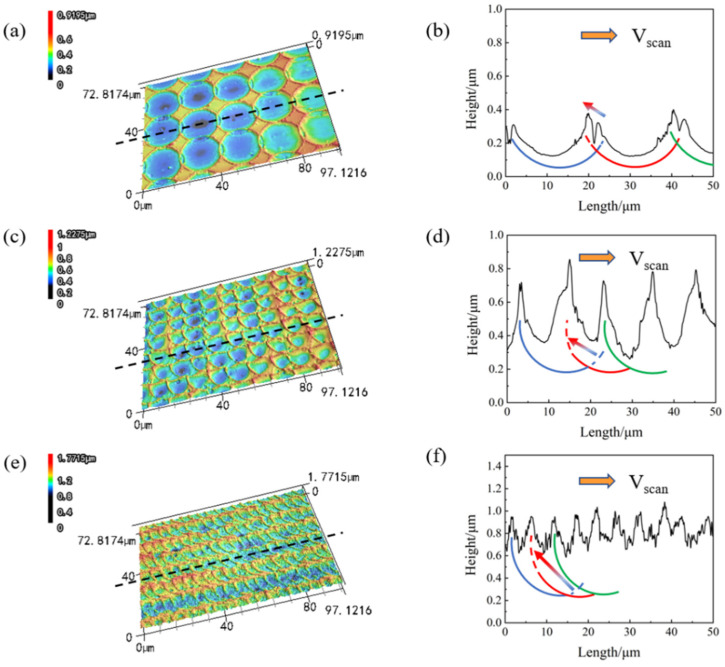
LCM image and cross-sectional view of the laser-textured glass at different scanning distances. (**a**,**b**) the scanning distance is 20 μm, (**c**,**d**) the scanning distance is 10 μm, (**e**,**f**) the scanning distance is 5 μm.

The morphology measurements are performed with the laser confocal microscope for laser-textured glass at different scanning distances. The LCM images and cross-sectional views under three different scanning distances are shown in Figure 7, where the black dotted line in the LCM images represents the selected cross-sectional position of the cross-sectional view. At different scanning distances, the overlapping of light spots is different, and the flow of materials by Marangoni convection is different. In Figure 7b,d, and f, the blue, red, and green arcs represent the successively generated light spots, and the blue-red gradient arrows represent the material flow between the two successively formed ridges. As shown in Figure 7a, when the scanning distance is 20 μm, the overlap between the light spots is small. For the formation of the red spot, the laser irradiation position is the unprocessed material and a small part of the blue ridge structures, as shown in Figure 7b. Therefore, the Marangoni convection will mainly drive the unprocessed material on the upper side of the red arc and a small part of the material at the blue ridge to cross the blue ridge to form a new red ridge. Therefore, there are two ridges with different heights where the spots overlap. As shown in Figure 7b, when the scanning distance is 10 μm, the overlap between the light spots is enhanced. For the formation of the red spot, since the laser irradiation position is on the right blue ridge, the blue ridge will be completely ablated. Thus, the Marangoni convection will drive all the materials at the blue ridge and some unprocessed materials on the right to form a new red ridge, as shown in Figure 7d. When the material flows to the left, the height difference promotes the Marangoni effect, so the left side of the red arc in Figure 7d eventually generates ridge structures higher than those in Figure 7b. When the scanning distance is 5 μm, the overlap between the spots is further enhanced, as shown in Figure 7e. For the formation of the red spot in Figure 7f, the laser irradiation position is the left part of the right blue ridge, so the ablation and flow of the material are similar to those in Figure 7d. The laser irradiation causes complete ablation of the right blue ridge, but the laser irradiation on the right unprocessed material decreases. At the same time, the height difference on the left side is smaller, so the height of the generated ridge structures is slightly lower than that in Figure 7d. 

## 4. Discussion

**Figure 8:**
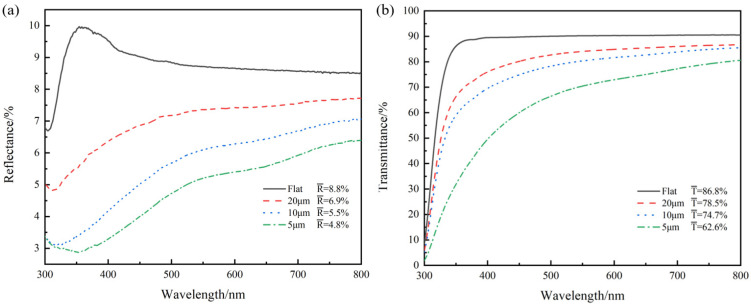
Reflectance and transmittance of unprocessed samples and laser-textured samples at different scanning distances. (**a**) the reflectance spectrum, and (**b**) the transmittance spectrum.

The multiscale structure obtained at different scanning distances differs in both ridge density and nanoparticle abundance. Figure 8a shows the reflection spectrum of the multiscale structural samples at three scanning distances, compared with the unprocessed samples. In Figure 8a, the multiscale structure has an excellent anti-reflection effect. With the decrease of the scanning distance, the anti-reflection effect is obviously improved by the increase of ridge structures and nanoparticles. When the scanning distance is 5 μm, the average reflectance of the laser-textured glass in the 300–800 nm band is 4.8%, while that of the unprocessed glass is 8.8%. Compared with the unprocessed sample, the reflectance of the multiscale structural sample is reduced by 45.5%, and it has excellent broadband anti-reflection capability. At the same time, the reflectance of the multiscale structural sample is reduced by 71.0% at the wavelength of 358 nm, which is meaningful for anti-reflection applications at specific wavelengths.

Figure 8b shows the transmission spectrum of the self-generated multiscale structural samples at three scanning distances. Compared with unprocessed glass, the average transmittance of laser-textured glass is decreased by 9.6%, 13.9%, and 27.8% when the scanning distances are 20 μm, 10 μm, and 5 μm, respectively. However, as seen from Figure 8a, the reflectance of laser-textured glass is decreased by 21.6%, 37.5%, and 45.5%, respectively, at three scanning distances. Transparent materials often have the problem of light transmittance reduction after surface processing. However, when the scanning spacing is 20 μm and 10 μm, the surface transmittance of multiscale structure can still be maintained at about 75%, which can meet various applications in daily life [16]. Meanwhile, the decreasing ratio of reflectance of multiscale structures is obviously higher than that of transmittance. In optical precision detection equipment, reflected light is often regarded as an interference signal. The proportion of interference signal in the transmission signal will obviously decrease by using laser textured glass instead of unprocessed glass.

**Figure 9:**
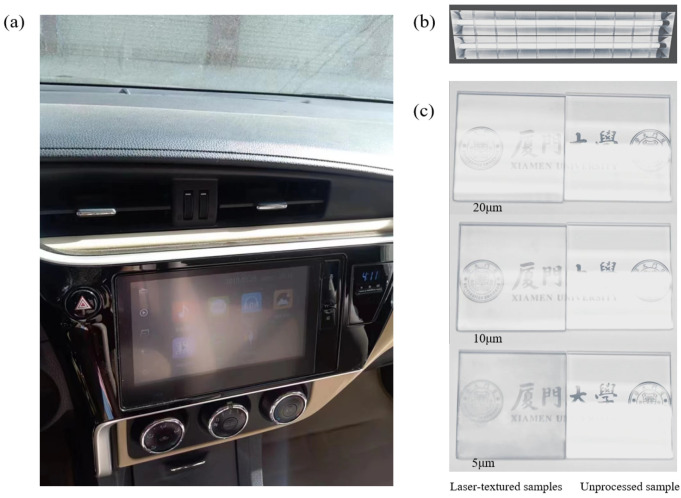
Glare phenomenon in automobile display equipment and anti-glare tests of laser textured samples. (**a**) glare phenomenon, (**b**) strong indoor light, and (**c**) anti-glare testing.

As the most used means of transportation in daily life, the safety performance of automobiles has attracted tremendous attention. In the process of driving, strong sunlight and other light may cause glare on the navigation screen, instrument panel, and windshield surface, which will affect the driver’s sight (Figure 9a). Laser-textured glass applied to display components of transportation tools can greatly reduce glare and improve driving safety. Indoor strong light (Figure 9b) is used to test the glare of glass in the face of strong light. Figure 9c shows a schematic of the anti-glare capability of the three laser-textured samples and the unprocessed sample. The numbers marked are the laser scanning distance of the laser-textured sample. When the unprocessed sample is exposed to strong light, the glare generated by the reflected light is obvious. The surface produces obvious bright stripes, and the characters and patterns on the lower side of the sample are completely unrecognizable. In contrast, when the laser-textured samples are faced with strong light, the width and number of bright stripes on the sample surface are obviously reduced. The glare is significantly diminished, and the characters and patterns on the lower side of the samples can be recognized. For the laser-textured samples, when the scanning distance is 20 μm, there are more bright stripes on the surface. As the scanning distance decreases, both the width and the number of bright stripes on the sample surface decrease, and the anti-glare ability of the laser-textured sample increases.

The line roughness R_a_ and R_z_, as well as the surface roughness S_a_ and S_z_, are used to evaluate the roughness of the surfaces, as shown in Table 1. The line roughness of the three samples met R_a_ 0.1 and R_z_ 0.8, reaching the N3 standard with a high degree of surface smoothness. The values of Sa and R_a_ are close to each other, and S_z_ is slightly larger than R_z_, indicating a high degree of uniformity in all directions of the sample surface and a small surface irregularity defect. This indicates that the laser-textured glass can maintain a high degree of surface smoothness.

## 5. Conclusions

In summary, a strategy for laser surface processing based on the Marangoni effect is proposed and experimentally demonstrated in this paper. This strategy is designed to prepare highly efficient anti-reflective and anti-glare structures on transparent material surfaces by a controlled single-step and chemical-free method to generate multiscale hybrid structures on the material surface. Firstly, simulation shows that multiscale structures have better anti-reflection performance than single-scale structures. After that, the influence of different laser parameters on the structures produced by the Marangoni effect and the laser-induced generation of nanoparticles are investigated. Finally, the laser-texturized sample with low reflectance is obtained when the energy density of the infrared femtosecond laser is 95.5 J/cm^2^ and the scanning distance is 5 μm. The average reflectance of this sample in the 300–800 nm band is reduced by 45.5% compared to the unprocessed glass. This sample also has anti-glare properties, and the surface can maintain high transmittance as well as high smoothness. Compared with the current common anti-reflection technology, the method is simple, environmentally friendly, low in cost, and easy to manufacture anti-reflection structures on a large scale. It is suitable for various types of optical windows, windshields, anti-glare glasses, electronic screens, instrument dials, and so on, which can work in strong light environments. This strategy is highly universal for materials and can be applied to the design of anti-reflection in metals and various flexible materials as well.

## Figures and Tables

**Table 1:** Roughness of samples with different scanning distances.

Scanning Distances	R_a_/(μm)	R_z_/(μm)	S_a_/(μm)	S_z_/(μm)
20 μm	0.0727	0.3785	0.0754	0.6370
10 μm	0.1176	0.6343	0.1099	0.8514
5 μm	0.0756	0.5458	0.0856	0.910

## Data Availability

The data presented in this study are available on request from the corresponding author.
